# Estrogen protects neuronal cells from amyloid beta-induced apoptosis via regulation of mitochondrial proteins and function

**DOI:** 10.1186/1471-2202-7-74

**Published:** 2006-11-03

**Authors:** Jon Nilsen, Shuhua Chen, Ronald W Irwin, Sean Iwamoto, Roberta Diaz Brinton

**Affiliations:** 1 Department of Pharmacology and Pharmaceutical Sciences, School of Pharmacy, University of Southern California, Los Angeles, California, 90033, USA; 2 Program in Neuroscience, University of Southern California, Los Angeles, California, 90033, USA

## Abstract

**Background:**

Neurodegeneration in Alzheimer's disease is associated with increased apoptosis and parallels increased levels of amyloid beta, which can induce neuronal apoptosis. Estrogen exposure prior to neurotoxic insult of hippocampal neurons promotes neuronal defence and survival against neurodegenerative insults including amyloid beta. Although all underlying molecular mechanisms of amyloid beta neurotoxicity remain undetermined, mitochondrial dysfunction, including altered calcium homeostasis and Bcl-2 expression, are involved in neurodegenerative vulnerability.

**Results:**

In this study, we investigated the mechanism of 17β-estradiol-induced prevention of amyloid beta-induced apoptosis of rat hippocampal neuronal cultures. Estradiol treatment prior to amyloid beta exposure significantly reduced the number of apoptotic neurons and the associated rise in resting intracellular calcium levels. Amyloid beta exposure provoked down regulation of a key antiapoptotic protein, Bcl-2, and resulted in mitochondrial translocation of Bax, a protein known to promote cell death, and subsequent release of cytochrome c. E_2 _pretreatment inhibited the amyloid beta-induced decrease in Bcl-2 expression, translocation of Bax to the mitochondria and subsequent release of cytochrome c. Further implicating the mitochondria as a target of estradiol action, *in vivo *estradiol treatment enhanced the respiratory function of whole brain mitochondria. In addition, estradiol pretreatment protected isolated mitochondria against calcium-induced loss of respiratory function.

**Conclusion:**

Therefore, we propose that estradiol pretreatment protects against amyloid beta neurotoxicity by limiting mitochondrial dysfunction via activation of antiapoptotic mechanisms.

## Background

A growing body of evidence supports the critical role of amyloid beta peptide (Aβ)^2 ^in Alzheimer's disease (AD) pathogenesis. Early onset AD is associated with overproduction of the 42-amino acid form of Aβ (Aβ_1–42_) and Aβ_1–42 _is toxic to neurons *in vitro *and *in vivo *[1-3]. To develop therapeutic strategies for reducing neuronal loss in AD, much effort has been extended to determine the molecular interactions underlying Aβ-induced neurotoxicity. Several lines of evidence suggest that calcium plays a key role in age-related changes in the brain that lead to AD and dementia [4-7]. Free intracellular calcium is a key activator of many signal transduction pathways of neurons, and alterations in intracellular calcium homeostasis are pivotal regulators of brain aging, memory and cell death [4-6,8-11]. According to a "calcium hypothesis" of AD, disturbances in calcium homeostasis are the proximal cause of neurodegeneration in AD, in which calcium dysfunction augments tau hyperphosphorylation, Aβ formation and neurotoxicity [4,5,9,12].

Calcium (Ca^2+^) transients in neurons are largely determined by mitochondria due to their large Ca^2+ ^capacity [13]. Mitochondrial Ca^2+ ^uptake is driven by the mitochondrial membrane potential (ΔΨ_m_) and occurs at the threshold of cytosolic Ca^2+^, followed by slow release, leading to a net accumulation of mitochondrial calcium ([Ca^2+^]_m_) and an alteration of physiological [Ca^2+^]_i _transients [14,15]. Mitochondrial dysfunction often leads to dysregulation of Ca^2+ ^homeostasis and consequent adverse downstream effects, including further damage to mitochondria setting up the mitochondrial spiral that is associated with multiple central nervous system disorders [16]. During aging, especially during the development and progression of neurodegenerative diseases, including AD, damaged mitochondria are unable to maintain the energy demands of the cell [17,18]. This can lead to an increased production of free radicals, which induces the interruption of oxidative phosphorylation, resulting in decreased levels of ATP that are necessary for normal energy homeostasis [16]. Apoptosis of degenerating neurons occurs in association with an accumulation of abnormal mitochondria in perikaryal regions and oxidative damage to the nucleus [19]. The same pattern of mitochondria lesions is observed in human AD brain biopsy samples [19]. Thus, therapeutic interventions that promote mitochondrial function could be a strategy to prevent neuronal dysfunction and death.

A prevention mode of estrogen exposure can prevent neurotoxicity both *in vitro *and *in vivo *[20-25]. Many of the proximate effects of estrogen treatment that have been associated with estrogen-induced neuroprotection converge upon the mitochondria [7,26]. We have previously shown that estrogen-induced neuroprotection is mediated through regulation of calcium homeostasis [22,26]. Further, we have shown that 17β-estradiol restores calcium homeostasis in neurons from middle-aged and old rats [27]. That the estrogen-induced regulation of calcium homeostasis is dependent upon maintenance of mitochondrial respiration and ΔΨ_m _[22] indicates a direct role of mitochondria in the neuroprotective effects of estrogen.

In the present study, we investigated the mechanisms whereby 17β-estradiol (E_2_) exposure can prevent Aβ-induced neuronal apoptosis and mitochondrial function. Results of these analyses indicate that E_2 _protects rat hippocampal neurons against Aβ toxicity and that such protection is associated with maintenance of calcium homeostasis, a decrease of cytochrome c release, a decrease of Bax translocation to the mitochondria and enhanced mitochondrial respiratory function. These data together with previously published findings indicate that E_2 _exposure prior to Aβ insult leads to a complex array of responses that coalesce in an organized cellular strategy to prevent loss of mitochondrial calcium homeostasis while simultaneously promoting Bcl-2 family protein strategies that prevent opening of the membrane permeability transition pore.

## Results

### 17β-estradiol prevents amyloid beta-induced neuronal death

**Figure 1:**
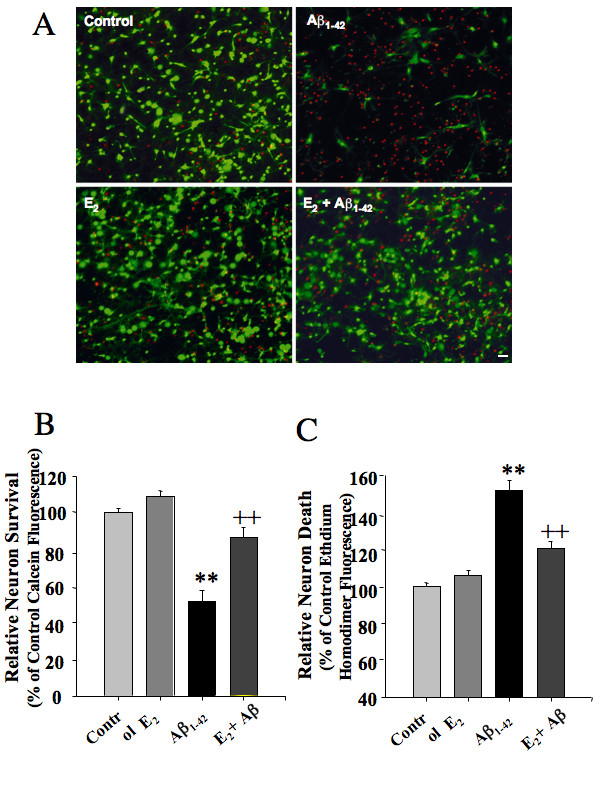
17β-estradiol prevents Amyloid beta_1–42_-induced neurotoxicity. Primary hippocampal neurons pretreated with E_2 _(10 ng/mL; 48 hr) or vehicle control were exposed to Aβ_1–42 _for 3 days and assessed for neuronal survival by calcein/ethidium homodimer staining. *A*, Visualization of viable (green) and dead (red) neurons. Bar = 20 μm. *B-C*, Fluorometer readings of calcein (live) and ethidium homodimer (dead) signals. (** = p < 0.01 as compared to control; ++ = p < 0.01 as compared to Aβ_1–42 _alone; n = 4).

To determine the impact of 17β-estradiol (E_2_) on Aβ neurotoxicity, 10 day old rat primary hippocampal neurons with elaborate neurite networks were treated with E_2 _(10 ng/mL) for 48 hr prior to exposure to fibrillar Aβ_1–42 _(1.5 μM) for 72 hr in the continued presence of E_2_. The concentration of 10 ng/ml of E_2 _was used based on multiple prior analyses indicating that this concentration induces maximal neuroprotection and the least degree of variability between experiments [20,26-28]. CalceinAM and ethidium homodimer staining, which reflect neuronal survival and death, respectively, indicated neuronal death following 72 hr exposure to Aβ_1–42 _(1.5 μM) compared to vehicle control-treated cultures (Fig. 1). Aβ_1–42 _exposure significantly decreased neuronal survival (Fig. 1B) as indicated by a decrease in the calcein signal (Fig. 1B; p < 0.01 as compared to control; n = 4) and an increase in the amount of ethidium homodimer signal (Fig. 1C; p < 0.01 as compared to control; n = 4). E_2 _(10 ng/mL) had no effect on neuronal survival in the absence of Aβ_1–42 _(Figure 1A,B &1C). Pretreatment of hippocampal neurons with E_2 _(10 ng/mL) significantly reduced the amount of Aβ_1–42_-induced neurotoxicity (Fig. 1A), as indicated by the significant attenuation of the Aβ_1–42_-induced neuronal death as indicated by increased Calcein fluorescence (Fig. 1B; p < 0.01 as compared to Aβ_1–42 _alone; n = 4) and decreased ethidium homodimer fluorescence (Fig. 1C; p < 0.01 as compared to Aβ_1–42 _alone; n = 4). Following 72 hr of Aβ exposure, neurites were thickened and beaded and there was an increase in the number of phase bright cells, indicative of neuronal death compared to vehicle control and E_2_-treated neurons (data not shown).

**Figure 2:**
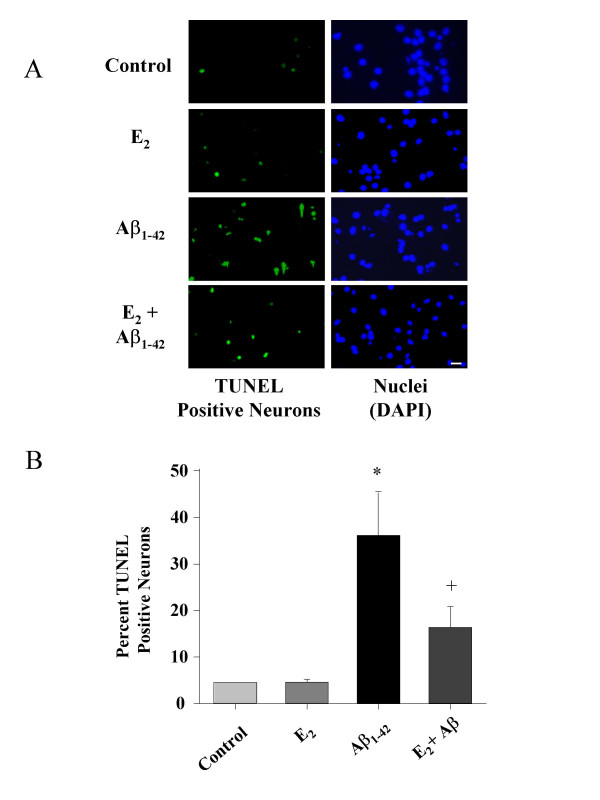
17β-estradiol prevents Amyloid beta_1–42_-induced apoptosis. Primary hippocampal neurons pretreated with E_2 _(10 ng/mL; 48 hr) or vehicle control were exposed to Aβ_1–42 _for 3 days and assessed for apoptosis by TUNEL staining. *A*, Representative images of TUNEL positive neurons and total nuclei. Bar = 20 μm. *B*, Counts of TUNEL positive neurons normalized to total nuclei from 3 random fields per well are presented as mean ± SEM. (* = p < 0.05 as compared to control; + = p < 0.05 as compared to Aβ_1–42 _alone; n = 4).

To determine if E_2_-induced alterations in neuronal survival were mediated via regulation of apoptosis, TUNEL staining was performed to identify apoptotic nuclei in response to Aβ_1–42 _in the presence and absence of E_2 _(Fig. 2A). Primary hippocampal neurons were pretreated with E_2 _(10 ng/mL) or vehicle control for 48 hr prior to Aβ_1–42 _(1.5 μM) exposure for 3 days in the continued presence of E_2 _or vehicle control. Aβ_1–42 _exposure resulted in a significant increase in the number of TUNEL-positive neurons (Fig. 2B; p < 0.05 as compared to control; n = 4). E_2 _treatment prior to Aβ_1–42 _exposure resulted in a significantly lower percentage of TUNEL-positive neurons than observed in response to Aβ_1–42 _exposure in vehicle control treated neurons, indicating that E_2_-treatment significantly reduced the amount of Aβ_1–42_-induced apoptosis (Fig. 2A &2B; p < 0.05 as compared to Aβ_1–42 _alone; n = 4). E_2 _alone had no significant effect on the percentage TUNEL-positive neurons (Fig. 2).

### 17β-estradiol prevents amyloid beta_1–42_-induced dysregulation of calcium homeostasis

**Figure 3:**
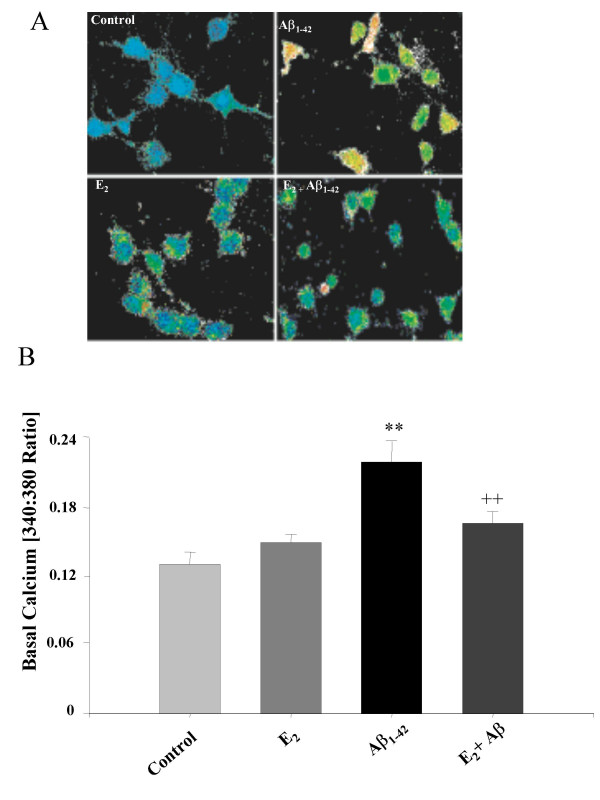
17β-estradiol prevents Amyloid beta_1–42_-induced rise in resting calcium concentration. Primary hippocampal neurons pretreated with E_2 _(10 ng/mL; 48 hr) or vehicle control were exposed to Aβ_1–42 _for 24 hr and assessed for cytosolic calcium concentration by Fura2 imaging. A. Representative images of Fura2 fluorescence in psuedocolor representing 340/380 ratio. B. Quantitative changes in Fura2 340/380 ratio presented as means +/- S.E.M. (** = p < 0.01 as compared to control; ++ = p < 0.01 as compared to Aβ_1–42 _alone; n = 4).

One potential mechanism of Aβ_1–42_-induced neurotoxicity is dysregulation of calcium homeostasis, which leads to an increased cytosolic calcium load and can result in neuronal dysfunction and death [4,9,27]. We have previously shown that estrogen-induced neuroprotection against glutamate excitotoxicity is mediated via regulation of neuronal calcium homeostasis [22] and that 17β-estradiol can both promote and restore calcium homeostasis in neurons derived from middle aged and aged rat hippocampi [27]. To determine if E_2_-induced neuroprotection against Aβ_1–42 _is likewise due to regulation of calcium homeostasis, we conducted Fura2 calcium imaging to assess the effect of Aβ_1–42 _on basal neuronal calcium loads in the presence and absence of E_2 _(Fig. 3). Hippocampal neurons were pretreated with E_2 _(10 ng/mL) or vehicle control for 48 hr prior to Aβ_1–42 _exposure for 24 hr. Aβ_1–42 _exposure resulted in a significant increase in the resting cytosolic calcium concentration (Fig. 3; p < 0.01 as compared to control; n = 4). E_2 _pretreatment significantly attenuated the Aβ_1–42_-induced rise in resting calcium concentration Fig. 3; p < 0.01 as compared to Aβ_1–42 _alone; n = 4). E_2 _by itself had no significant effect on resting calcium concentration (Fig. 3).

### 17β-estradiol prevents amyloid beta_1–42_-induced depletion of mitochondrial Bcl-2

**Figure 4:**
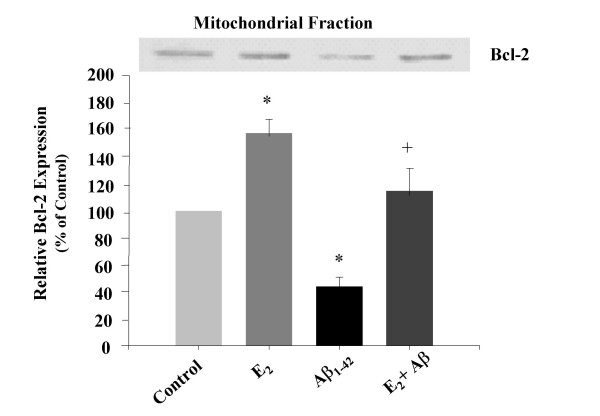
17β-estradiol prevents Amyloid beta_1–42_-induced decline in mitochondrial Bcl-2 expression and Bax translocation. Primary hippocampal neurons pretreated with E_2 _(10 ng/mL; 48 hr) or vehicle control were exposed to Aβ_1–42 _for 24 hr and mitochondrial fractions were assessed for (*A*) Bcl-2 and (*B*) Bax expression by Western blot analysis. (* = p < 0.05 as compared to control; ** = p < 0.01 as compared to control; + = p < 0.05 as compared to Aβ_1–42 _alone; n = 4).

Bcl-2 is well established as an anti-apoptotic protein in neurons that can avert neuronal death and protect mitochondria against toxin-induced dysfunction [29,30]. It has been shown that Aβ down regulates Bcl-2 in human primary neurons [31]. Further, Bcl-2 is an estrogen-responsive protein [7,22,32-35]. It has also been suggested that Bcl-2 is involved in maintenance of cellular calcium homeostasis [29,30]. To determine if mitochondrial expression of this key regulator of apoptosis was involved in E_2_-induced neuroprotection, we determined the effect of E_2 _and Aβ_1–42 _on Bcl-2 expression in the mitochondrial fraction of rat primary hippocampal neurons. Neurons were pretreated with E_2 _(10 ng/mL) or vehicle control for 48 hr prior to exposure to Aβ_1–42 _(1.5 μM) for 24 hr. Crude mitochondrial fractions were assessed for Bcl-2 expression by Western blot analysis. Exposure to Aβ_1–42 _resulted in a significant decrease (~60%) in Bcl-2 expression in the mitochondrial fraction (Fig. 4; p < 0.01 as compared to control; n = 4). As expected, E_2 _by itself significantly increased (~60%) mitochondrial Bcl-2 expression (Fig. 4A; p < 0.05 as compared to control). Pretreatment of neurons with E_2 _completely prevented Aβ_1–42_-induced decrease in mitochondrial Bcl-2 (Fig. 4; p < 0.01 as compared to Aβ_1–42 _alone; p < 0.05 as compared to control; n = 4).

### 17β-estradiol prevents amyloid beta_1–42_-induced translocation of Bax

Neurotoxicity resulting from apoptosis often results from the induction of Bax translocation from the cytosol to the mitochondria where it can mediate the release of apoptotic factors such as cytochrome c [36-39]. To determine if the neuroprotective effect of E_2 _is associated with regulation of Bax translocation or cytochrome c release, primary hippocampal neurons were pretreated with E_2 _(10 ng/mL) or vehicle control for 48 hr prior to Aβ_1–42 _(1.5 μM) exposure for 24 hr and assessed for Bax localization by Western blot and immunocytochemical analyses.

**Figure 5:**
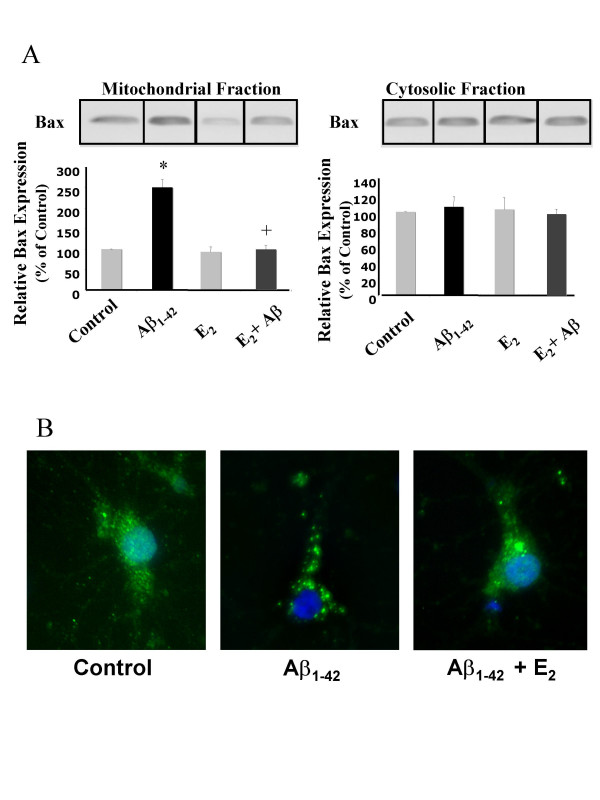
17β-estradiol prevents Amyloid beta_1–42_-induced Bax translocation. Primary hippocampal neurons pretreated with E_2 _(10 ng/mL; 48 hr) or vehicle control were exposed to Aβ_1–42 _for 24 hr and mitochondrial fractions were assessed for Bax expression by (A) Western blot analysis and (B) immunocytochemistry. The order of bands was switched to maintain a consistent treatment order across all figures for presentation purposes only. All quantitation and analysis was performed unmodified images. (* = p < 0.05 as compared to control; ** = p < 0.01 as compared to control; + = p < 0.05 as compared to Aβ_1–42 _alone; n = 4).

Aβ_1–42 _exposure resulted in a significant increase in Bax immunoreactivity in the mitochondrial fraction (Fig. 5; p < 0.05 as compared to control; n = 4). E_2 _pretreatment significantly attenuated the Aβ_1–42_-induced increase in Bax immunoreactivity in the mitochondrial fraction (Fig. 5A; p < 0.05 as compared to Aβ_1–42 _alone; n = 4). In contrast to the mitochondrial fraction there was no change in Bax immunoreactivity in the cytosolic fraction (Fig. 5A; n = 4). To confirm the shift in subcellular localization of Bax, we performed immunoflourescent staining for Bax. In control neurons, the Bax immunofluorescence signal was diffuse throughout the cell body and neurites (Fig. 5B, left panel). In Aβ_1–42_-treated cells the Bax immunofluorescence signal was punctate, consistent with a shift to mitochondrial localization (Fig. 5B middle panel). Pretreatment of hippocampal neurons with E_2 _prevented Aβ_1–42_-induced translocation of Bax, resulting in a diffuse cytosolic staining pattern with slight areas of clustering consistent with partial mitochondrial translocation (Fig. 5B, right panel).

### 17β-estradiol prevents amyloid beta_1–42_-induced release of cytochrome c

**Figure 6:**
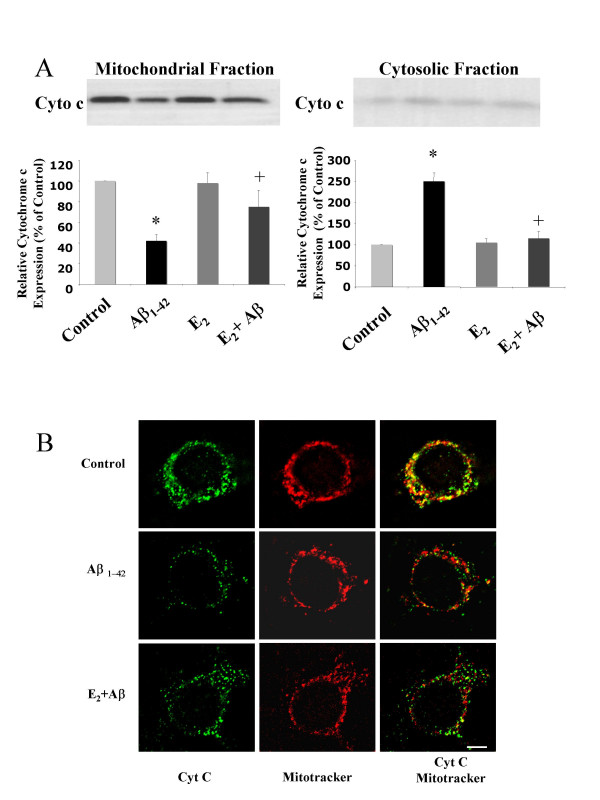
17β-estradiol prevents Amyloid beta_1–42_-induced cytochrome c release. Primary hippocampal neurons pretreated with E_2 _(10 ng/mL; 48 hr) or vehicle control were exposed to Aβ_1–42 _for 24 hr and assessed for cytochrome c expression by Western blot analysis and immunocytochemistry. *A*, Western blot analysis cytochrome c expression in cytosolic (right panel) and mitochondrial (left panel) fractions of treated neurons. *B*, Treated neurons were immunostained for cytochrome c expression (Green) and mitochondria were labeled with Mitotracker Red CMXRos (Red). Co-localization of cytochrome c with mitochondria is visible in the overlay image (yellow). Bar = 5 μm.

To determine the consequences of regulation of Bcl-2 and translocation of Bax, we determined the impact of E_2 _pretreatment and Aβ_1–42 _exposure on cytochrome C release from mitochondria. Aβ_1–42 _exposure resulted in a significant increase in cytochrome c immunoreactivity in the cytosolic fraction (Fig. 6A; p < 0.05 as compared to control; n = 4). E_2 _pretreatment significantly attenuated the Aβ_1–42_-induced increase in cytochrome c immunoreactivity in the cytosolic fraction while having no effect alone (Fig. 6A; p < 0.05 as compared to Aβ_1–42 _alone; n = 4). There was a corresponding significant decrease in cytochrome c immunoreactivity in the mitochondrial fraction in response to Aβ_1–42 _(Fig. 6A; p < 0.05 as compared to control; n = 4) that was prevented by E_2 _pretreatment (Fig. 6A; p < 0.05 as compared to Aβ_1–42 _alone; n = 4). To confirm the subcellular localization of cytochrome c in our conditions, neurons were double stained for cytochrome c and mitochondria (Mitotracker Red CMXRos). In control neurons cytochrome c immunofluorescence (green) exhibited a punctate staining pattern that overlapped (yellow) with the mitochondrial localization signal (red) (Fig. 6B). Aβ_1–42 _exposure resulted in a diminution of the punctuate labeling of cytochrome c immunofluorescence (green) particularly in the soma while the sparse neuritic labeling for cytochrome C labeling remained. However, the neuritic labeling for cytochrome C was not co-localized with the mitochondrial localization signal (red) indicating release of cytochrome c from the mitochondria (Fig. 6B). The cytochrome c immunofluorescence (green) in the E_2 _pretreated and Aβ_1–42 _exposed neurons exhibited a punctate staining pattern that overlapped (yellow) with the mitochondrial localization signal (red), as in the control cultures (Fig. 6B).

**Figure 7:**
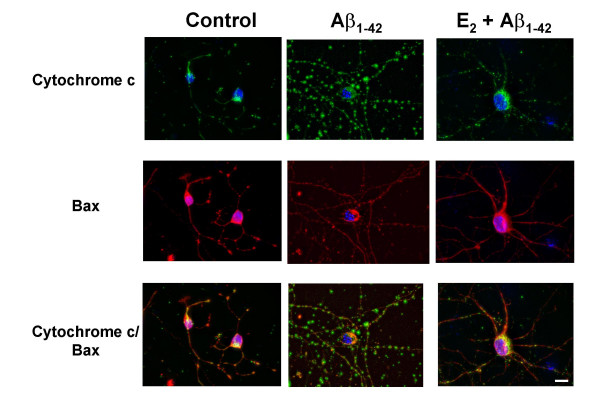
17β-estradiol prevents Amyloid beta_1–42_-induced Bax translocation and cytochrome c release. Primary hippocampal neurons pretreated with E_2 _(10 ng/mL; 48 hr) or vehicle control were exposed to Aβ_1–42 _for 24 hr and assessed for Bax and cytochrome c expression by immunocytochemistry. Treated neurons were double immunostained for Bax (Red) and cytochrome c expression (Green). Bar = 10 μm.

To determine the relationship between cytochrome c release and Bax translocation, hippocampal neurons were assessed for Bax and cytochrome c expression by double immunofluorescence (Fig. 7). In control neurons the Bax immunofluorescence signal (red) was diffuse and, as above, the cytochrome c immunofluorescence signal (green) was punctate (Fig. 7), consistent with cytosolic Bax and mitochondrial cytochrome c. In Aβ_1–42_-treated cells the Bax immunofluorescence signal (red) was punctate, consistent with a shift to mitochondrial localization, and, as above, the cytochrome c immunofluorescence signal (green) was diminished with a diffuse localization (Fig. 7). Pretreatment of hippocampal neurons with E_2 _prevented Aβ_1–42_-induced translocation of Bax and release of cytochrome C from mitochondria (Fig. 7).

### 17β-estradiol prevents neurotoxic-induced decline in mitochondria respiration

**Figure 8:**
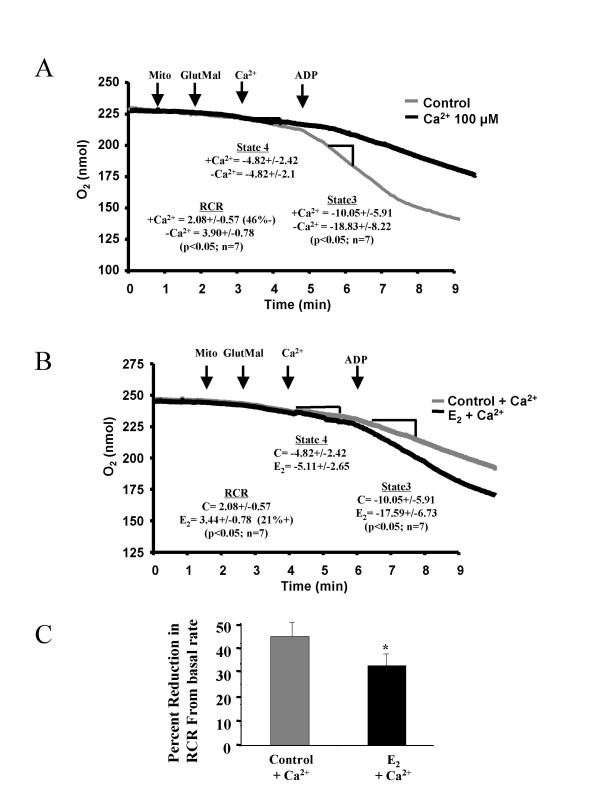
17β-estradiol prevents calcium-induced damage to mitochondrial respiratory function. A. Whole brain mitochondria were isolated from ovariectomized rats and assessed for mitochondrial respiration in the presence of glutamate (5 μM) and malate (5 μM) as respiratory substrates following a 2 min challenge with 100 μM Ca^2+ ^or buffer control. Respiratory control ratio (RCR) is the ratio of State 3 (ADP stimulated) respiration to State 4 (resting) respiration. (n = 7; * = p < 0.05) B. Whole brain mitochondria were isolated from ovariectomized rats treated with 17β-estradiol (30 μg/kg) or vehicle control for 24 hr and assessed for mitochondrial respiration following 2 min calcium (10 μM) exposure in the presence of glutamate (5 μM) and malate (5 μM) as respiratory substrates. C. Respiratory control ratio (RCR) is the ratio of State 3 (ADP stimulated) respiration to State 4 (resting) respiration. (n = 7; * = p < 0.05)

We sought to determine if the E_2 _treatment that prevented the Aβ_1–42_-induced alterations in mitochondrial-associated proteins of primary hippocampal neurons affected brain mitochondria *in vivo*. An increased mitochondrial calcium capacity should coincide with an enhanced ability to withstand calcium load and in parallel a sustained mitochondrial respiratory function. Whole brain mitochondria were isolated and assessed for respiratory function in the presence of glutamate and malate as respiratory substrates following a 2 min challenge with 100 μM Ca^2+^. Oxygen consumption during State 4 (resting) respiration was not affected by the presence or absence of calcium (100 μM) in control mitochondria (Fig. 8A). Although mitochondrial calcium overload can result in an uncoupling effect that would be expected to increase State 4 respiration, this was relatively unchanged in these experiments, consistent with previously published results [30]. Oxygen consumption during State 3 (ADP stimulated) respiration was significantly decreased following Ca^2+ ^challenge in control brain mitochondria relative to Ca^2+ ^naive mitochondria (Fig. 8A; p < 0.05; n = 7). There was a likewise decrease (~45%) in the respiratory control ratio (RCR) following Ca^2+ ^challenge of control mitochondria (Fig. 8C; p < 0.05; n = 7). RCR values determined using atractyloside to induce State 4 respiration following ADP depletion were indistinguishable from those calculated using basal State 4 respiration readings (data not shown).

To determine if E_2_-treatment serves a protective function against increased calcium loads, mitochondrial respiratory function was assessed following a 2 min calcium exposure in the presence of glutamate and malate. Ovariectomized adult rats were administered E_2 _(30 μg/kg in sesame oil) or vehicle control (sesame oil) 24 hr prior to mitochondrial isolation from whole brain tissue. Brain mitochondria from E_2_-treated rats displayed significantly greater oxygen consumption following Ca^2+ ^challenge in State 3 respiration and a significantly higher RCR than mitochondria from control rats (Fig. 8C; p < 0.05; n = 7). Further there was a smaller decrease in the RCR values following Ca^2+ ^challenge in the brain mitochondria from E_2_-treated rats than control (Fig. 8C; p < 0.05; n = 7).

## Discussion

Neurodegenerative diseases, like Alzheimer's disease, are associated with disruption of calcium homeostasis and mitochondrial dysfunction leading to apoptotic events and neuronal cell death. Previously we demonstrated that 17β-estradiol protects against age-related calcium dysregulation [27]. In this study we show that estrogen pretreatment prevents amyloid beta-induced calcium dysregulation, mitochondrial failure and resultant apoptosis in hippocampal neuronal cultures. These results are consistent with previous data that demonstrate that estrogen is neuroprotective against Aβ-induced neurotoxicity [21,27,40], but further the field by providing evidence for the underlying mechanism of E_2_-induced neuroprotection through regulation of mitochondrial signals that initiate and activate apoptotic processes.

During aging, especially during the development and progression of neurodegenerative diseases, including Alzheimer's disease (AD), damaged mitochondria are unable to maintain the energy demands of the cell. Interrupted energy metabolism is observed in many instances of neurodegeneration [16,18,19,41,42], including cerebral ischemia and Alzheimer's disease [18], two neurological conditions that account for the majority of all neurodegenerative conditions. Reductions in cerebral metabolic rate often occur before the development of clinical disabilities. Impairment of oxidative energy metabolism leads to increased expression of amyloid precursor protein (APP) [16] and to cytoskeletal disorganization, including the appearance of epitopes associated with paired helical filaments/tangles [43,44]. This can lead to an increased production of free radicals, which induces the interruption of oxidative phosphorylation, resulting in decreased levels of ATP that are necessary for normal energy homeostasis. Subsequently, cerebral metabolism may be unable to meet the energy demands required to restore dissipated membrane potentials, as ATP in the injured brain is significantly reduced. Furthermore, a limited energy supply is evident from the reductions in mitochondrial oxygen consumption, mitochondrial membrane potential, and mitochondrial enzymatic activity. In this study we demonstrated that E_2 _prevented the neurotoxic-induced decline in mitochondrial respiratory function. This is consistent with the previous reports that E_2 _is protective against cell death induced by energy depletion [45] and blocks the decrease in mitochondrial membrane potential induced by Aβ in PC12 cells [46]. Thus E_2 _may be able to prevent interruption of neuronal energy metabolism associated with neurodegeneration and may reduce APP expression and paired helical filament formation.

Underlying the neurodegeneration-associated mitochondrial dysfunction appears to be dysregulation of calcium homeostasis. Several lines of evidence suggest that calcium plays a key role in age-related changes in the brain that lead to AD and dementia. Free intracellular calcium is one of the most important messengers for many signal transduction pathways of neurons, and alterations in intracellular calcium homeostasis are critically involved in brain aging, memory and cell death. Landfield's group showed that altered intracellular calcium is directly correlated with impaired neuronal plasticity such that elevated intracellular calcium and frequency facilitation were negatively correlated in individual old neurons within hippocampal slices [47]. This finding led these investigators to postulate that intracellular calcium is likely elevated in old hippocampal neurons and frequency facilitation would thus be impaired in old hippocampal neurons during the theta frequencies associated with cognitive processing. Consistent with this postulate are recent *in vivo *data from studies in old rats. Foy and colleagues found that E_2 _suppressed the calcium-dependent induction of long-term depression in CA1 hippocampal neurons of old rats [48]. A later analysis by Foster and co-workers showed that E_2 _decreased the Ca^2+^-activated afterhyperpolarization which is larger in old rats compared to young rat CA1 neurons and is enhanced by a higher density of L-type calcium channels in the old rat neuron [49]. More recently we have shown that estrogen can reverse the age-associated calcium dysregulation in primary neuronal cultures obtained from the hippocampus of aged rats [27].

According to a "calcium hypothesis" of AD, arising from numerous preclinical *in vitro *studies, disturbances in calcium homeostasis are the proximal cause of neurodegeneration in AD [9]. There is a large body of evidence from preclinical experimental models and from human subjects that alterations in calcium signalling occur during initial phases of AD, even before the development of overt symptoms or any obvious extracellular amyloid-beta pathology [4]. In the current studies, we showed that E_2 _pretreatment prevented the Aβ-induced rise in resting calcium concentration in primary hippocampal neurons, an effect similar to the E_2_-mediated reduction in calcium rise induced by excitotoxic glutamate [22,26]. More recently we have shown that estrogen can reverse the age-associated calcium dysregulation in primary neuronal cultures obtained from the hippocampus of aged rats [27]. A mitochondrial site of action for estrogen-mediated neuroprotection is supported by the functional mitochondria dependence of the attenuation of the glutamate-induced Ca^2+ ^rise [22].

One mechanism by which estrogen may regulate mitochondrial calcium homeostasis and maintain energy metabolism is via the antiapoptotic protein Bcl-2. The magnitude of Ca^2+ ^accumulation by mitochondria can be altered by Bcl-2 [29,30], which is localized to the mitochondrial membrane and its expression has been shown to significantly enhance mitochondrial Ca^2+ ^sequestration [29,30,50]. In the current studies we demonstrated that E_2 _increases, and prevents the Aβ-induced decrease in, the mitochondrial expression of Bcl-2, consistent with previous reports of E_2_-induced increases in Bcl-2 in reproductive tissues and brain [22,32-35,51]. In addition to increasing the magnitude of Ca^2+ ^sequestered by mitochondria, Bcl-2 enhances the tolerability of mitochondria for increased levels of [Ca^2+^]_i _that otherwise result in dissipation of ΔΨ_m _and cell death [52]. Consistent with the increase in mitochondrial Ca^2+ ^load tolerability demonstrated here, Mattson *et al*. showed that a supraphysiological concentration of E_2 _(10 μM) can preserve ΔΨ_m _in PC12 cells expressing mutant presenilin that were exposed to Aβ [46]. We propose that by increasing [Ca^2+^]_m _uptake capacity, and the Bcl-2-induced resistance to Ca^2+^-induced respiratory inhibition, E_2 _prevents Bax translocation and cytochrome c release, limiting the loss of viability initiated by neurotoxic insults.

## Conclusion

In summary, we have shown that E_2 _provides neuroprotection in rat hippocampal neurons subjected to Aβ toxicity and that such protection is associated with maintenance of calcium homeostasis, a decrease of cytochrome c release, a decrease of Bax translocation to the mitochondria and enhanced mitochondrial respiratory function. Taken together these data indicate that mechanisms of estrogen-inducible neuroprotection against degenerative insults are a function of estrogen activation of cellular mechanisms whose ultimate outcome is promotion of mitochondrial viability. Thus mitochondria are ideal therapeutic targets of estrogen and estrogen-like surrogates in brain. Further elucidation of the mitochondrial sites of estrogen action will allow for development of selective therapeutic agents for use in estrogen therapy for prevention of neurodegenerative diseases.

## Methods

### Chemicals

Culture materials were from Gibco BRL (Rockville, MD). Chemicals were from MP Biomed (Irvine, CA) unless otherwise noted. Steroids were dissolved in ethanol and diluted in culture medium with final ethanol concentration <0.001%. Fura2-AM, Calcein AM and ethidium homodimer-1 were from Molecular Probes (Eugene, OR). Amyloid beta_1–42 _was from American Peptide Company (Sunnyvale, CA).

### Neuronal culture

Hippocampal neurons from embryonic day 18 (E18) rat fetuses were cultured as previously described and generated cultures 98% neuronal in phenotype [22]. Briefly, embryonic rat hippocampi were dissociated by passage through fire-polish constricted Pasteur pipettes. Neurons plated on poly-d-lysine coated coverslips (22 mm round), chamberslides (Falcon; 4 well) or polyethylenimine coated 6-well plates were grown in Neurobasal medium supplemented with 5 U/ml penicillin, 5 mg/ml streptomycin, and B27 supplement at 37°C in humidified 5% CO_2 _atmosphere for 10–12 days prior to experimentation.

### Amyliod beta-induced injury

Neurons were pretreated with 17β-estradiol (10 ng/mL) or vehicle control for 48 hr prior to exposure to fibrillar amyloid beta (1.5 μM) for the indicated times. Fibrillar Aβ_1–42 _was prepared by solubilizing Aβ_1–42 _in HCL (10 mM) at a concentration of 1 mM. Aβ_1–42 _was diluted in PBS to 100 μM and incubated for 72 hr at 22°C [40]. At the beginning of each experiment Aβ_1–42 _was further diluted to 1.5 μM in complete Neurobasal medium.

### Neuronal survival

Cell viability was measured by calcein/ethidium homodimer staining. Neurons were incubated in phosphate buffered saline (PBS) containing calcein AM (1 μM) and ethidium homodimer-1 (2 μM) for 30 min at room temperature. Fluorescent intensity was measured using a dual-wavelength fluorescent plate reader (GeniosPro; Molecular Devices) at 485/530 nm Ex/Em and 530/645 nm Ex/Em for calcein and ethidium, respectively. Data represents percent live or dead cells normalized to control fluorescent values. Data is presented as mean ± S.E.M. from 4 independent experiments with 8 wells per condition per experiment. Images were captured using the Marianas imaging system with Slidebook software (Intelligent Imaging Innovations, Inc., Santa Monica, CA) based on a Zeiss 200 M inverted microscope.

### TUNEL staining

Neurons grown on chamber slides were treated as for Neuronal Survival above and fixed in 4% paraformaldehyde for 15 at room temperature and processed for TUNEL staining by the In Situ Cell Death Detection Kit, Fluorescein kit (Roche Applied Science; Indianapolis, IN) according to the manufacturer's instructions. Neurons were counterstained with DAPI to label nuclei. Three random fields per well were captured using the Marianas imaging system with Slidebook software (Intelligent Imaging Innovations, Inc., Santa Monica, CA) based on a Zeiss 200 M inverted microscope. Images were captured from 3 wells per condition per experiment (total of 9 fields per condition). Number of TUNEL positive neurons and number of nuclei (DAPI) were determined using Slidebook software and percent TUNEL positive neurons was calculated from the ratio of number of TUNEL positive neurons to number of total nuclei. Data represents the mean ± SEM from 4 independent experiments.

### Measurement of cytoplasmic Ca^2+ ^using Fura2-AM

Hippocampal neurons grown on glass coverslips were pretreated with E_2 _(10 ng/mL) or vehicle control for 48 hr prior Aβ_1–42 _exposure for 24 hr. Neurons were loaded in the dark with Fura2-AM (2 μM) in Hank's Buffered Saline (HBS) (45 min.; 37°C). Cytosolic calcium concentrations were determined by comparing the 340/380 ratio to a standard curve as previously described [22]. At least 10 neurons per coverslip were assessed with at least 3 coverslips per condition per experiment. Data represents the mean ± SEM from 4 independent experiments. Equal dye loading was determined as previously described [22].

### Western blot analysis

Cytosolic and mitochondrial fractions were obtained using the Cytosol/Mitochondrial Fractionation Kit (Calbiochem; Sand Diego, CA) according to the manufacturer's instructions. Protein concentration was determined by the BCA assay. 25 μg of total protein were loaded per well and electorphoresed in a 2% SDS-PAGE gel. Protein was electrotransfered to PVDF membranes and probed with primary antibodies to Bcl-2 (1:250; BD Transduction Laboratories (610539); San Jose, CA), Bax (1:1000; Cell Signaling Technology (2772); Beverly, MA) and cytochrome c (1:1000; BD Transduction Laboratories (5564333); San Jose, CA) and respective horseradish peroxidase (HRP)- conjugated secondary antibodies (1:10,000; 1 hr room temperature). Porin (Mitosciences, Eugen, OR) and Actin (Santa Cruz, Santa Cruz CA) were used as loading controls for mitochondrial and cytosolic fraction, respectively (Data not shown). Bands were visualized with TMB peroxidase kit (Vector Laboratories) and quantitated by scanning and densitometry with Un-Scan-It software (Silk Scientific; Orem, UT). Data are presented as means ± S.E.M. from 4 independent experiments.

### Immunostaining

Treated neurons grown on chamberslides were fixed in 4% paaraformaldehyde for 15 at room temperature 24 hr following Aβ exposure. For mitochondrial labeling, neurons were incubated in Mitotracker Red CMXRos for 10 min at 37°C for 10 min prior to fixation. Neurons were washed in PBS and permeabilized in PBS + 0.01% triton X-100 for 5 min prior to incubation in primary antibody (Bax: 1:250; Cell Signaling Technology (2772); Beverly, MA; cytochrome c: 1:250; Pharmingen (556432); San Jose, CA) for 2 hr at room temperature. Antibody-antigen complexes were visualized by incubating in FITC (1:250; Vector Laboratories; Burlingame, CA)- or CY3 (1:1000; Amersham; Piscataway, NJ)-conjugated secondary antibodies for 45 min at room temperature, coverslipping with DAPI containing mounting medium (Vector Laboratories; Burlingame, CA) and capturing images using the Marianas imaging system with Slidebook software (Intelligent Imaging Innovations, Inc., Santa Monica, CA).

### Mitochondrial isolation

Adult (4–6 months old) female ovariectomized Sprague-Dawley rats were injected subcutaneously with 17b-estradiol (30 μg/kg) in sesame oil 2 weeks following surgery. 24 hr later, rats were sacrificed and whole brain tissue was homogenized in mitochondrial isolation buffer (0.32 M sucrose, 1 mM EDTA and 10 mM Tris; pH 7.4). Homogenates were centrifuged at 1,330 × g for 5 min at 4°C. Pellets were re-homogenized and centrifuged. The two postnuclear supernantants were combined and centrifuged at 21,200 × g for 10 min at 4°C. The resulting crude mitochondrial pellets were resuspended in 15% Percoll and layered over a 23%/40% discontinuous Percoll gradient and centrifuged at 27, 000 × g for 10 min at 4°C. The fraction accumulating at the 23%/40% interface was collected and diluted 1:4 with isolation buffer and centrifuged at 16,000 × g for 10 min at 4°C.

The pellet was transferred to a 1.5 ml tube centrifuged at 7,300 × g for 10 min at 4°C. The pellet will be resuspended in 40 μL isolation buffer. Protein concentration will be determined by the BCA assay. Mitochondrial integrity was assessed assessing cytochrome c oxidase activity (Cytochrome c oxidase activity kit, Sigma) in intact and lysed mitochondrial samples. Cytochrome c oxidase in inaccessible in intact mitochondria and high activity in these samples relative to total activity (lysed samples) indicates poor mitochondrial integrity.

### Measurement of respiration in mitochondria isolated from rat brains

Aliquots of mitochondria (1 mg/mL) were used in measurements of respiratory activity using a Clark-type oxygen electrode (Hansatech Oxygraph) as previously described. Oxygen electrode buffer (130 mM KCl, 2 mM KH_2_PO_4_, 3 mM HEPES, 2 mM MgCl_2_, 1 mM EGTA) was incubated for 1 min in a magnetically stirred chamber at 28°C. The respiratory substrates, glutamate (5 mM) and malate (2.5 mM), were added followed by the isolated mitochondria (100 μg). Basal respiration was first measured in the absence of ADP for 1 min, followed by Ca^2+ ^(100 μM) or equivalent volume of buffer for an additional 2 min. Subsequently, State 3 respiration was measured in the presence of ADP, by addition of 20 mL ADP (0.027 M in 0.067 M NaPO_4_) to determine the maximal rate of coupled ATP synthesis. Then State 4 respiration was induced by addition of the adenine nucleotide translocator inhibitor atractyloside (50 μM). The respiratory control ratio was calculated using the ratio of State 3 to State 4 respiratory rates.

### Statistics

Statistically significant differences were determined by one-way ANOVA followed by Student-Neuman Keuls post hoc analysis.

## Abbreviations

The abbreviations used are: Aβ, amyloid beta; AD, Alzheimer's disease; Aβ_1–42_, Amyloid beta 1–42; Ca^2+^, calcium; ΔΨ_m_, mitochondrial membrane potential; [Ca^2+^]_m_, mitochondrial calcium concentration; [Ca^2+^]_i_, cytosolic calcium concentration; E_2_, 17β-estradiol; APP, amyloid precursor protein.

## Authors' contributions

JN conceived of the study, and participated in its design and coordination and drafted the manuscript. SC participated in the study design and carried out the cell culture, neuroprotective experiments and Western blot analyses. RWI performed the mitochondrial isolations and the respiratory studies. SI participated in the mitochondrial isolations and Western blot analyses. RDB participated in the study design and coordination and helped to draft the manuscript. All authors read and approved the final manuscript.
